# Supplementary material for: MetaQTL: a package of new computational methods for the meta-analysis of QTL mapping experiments
Source: BMC Bioinformatics. 2007 Feb 8;8:49. doi: 10.1186/1471-2105-8-49 (PMC1808479; doi:10.1186/1471-2105-8-49)
Supplement: Additional File 7 — MetaQTL Package : jar file and tutorial. This Zip archive contains both the MetaQTL JAR file and the files of the tutorial. [file 1471-2105-8-49-S7.zip › org.inra.metaqtl/doc/org/metaqtl/MapCluster.html]

MapCluster


|  |  |  |
| --- | --- | --- |
| **Overview** \| **Package** \| **Class** \| **Use** \| **Tree** \| **Deprecated** \| **Index** \| **Help** | |  |
| **PREV CLASS**   **NEXT CLASS** | **FRAMES**    **NO FRAMES**     **All Classes** |  |
| SUMMARY: NESTED \| FIELD \| CONSTR \| METHOD | DETAIL: FIELD \| CONSTR \| METHOD |  |


---


## org.metaqtl Class MapCluster

```
java.lang.Object
  org.metaqtl.MapCluster
```

---

``` public class MapCluster extends java.lang.Object ```

---

**Constructor Summary**

`MapCluster()`
  
          Creates a new instance of `MapCluster`.


| **Method Summary** | |
| --- | --- |
| `void` | `addAllMap(IBioGenome[] maps)` |
| `void` | `addChromosome(IBioLGroup group, IBioCross crossDesign, int mappingFunction, int mappingUnit, boolean rescale, boolean skeleton)`             Add a chromosome to the map cluster framework. |
| `void` | `addMap(IBioGenome map, boolean skeleton)`             Add a map to the cluster. |
| `java.lang.String` | `getChromosomeName(java.lang.String name)`             This method returns the name of the chromosome from the given name. |
| `ChromCluster[]` | `getClusters()`             Returns the chromosome clusters. |
| `MetaDico` | `getMarkerDico()` |
| `void` | `setDubiousMarker(IBioLocus[] dubious)`             This method sets the list of dubious marker. |
| `void` | `setMarkerDico(MetaDico mrkDico)` |

**Methods inherited from class java.lang.Object**

`clone, equals, finalize, getClass, hashCode, notify, notifyAll, toString, wait, wait, wait`

**Constructor Detail**

### MapCluster

```
public MapCluster()
```

:   Creates a new instance of `MapCluster`.


**Method Detail**

### addMap

```
public void addMap(IBioGenome map,
                   boolean skeleton)
```

:   Add a map to the cluster. This automatically classifies
    the chromosomes of the given map into clusters of chromosome
    using their names.

    :   **Parameters:**: `map` - the map to add.: `skeleton` - true if the chromosomes of this map must flagged as the skeleton chromosomes. **See Also:**: `ChromCluster`, `ChromCluster.addChromosome(IBioLGroup, IBioCross, int, int, boolean, boolean)`

---


### addChromosome

```
public void addChromosome(IBioLGroup group,
                          IBioCross crossDesign,
                          int mappingFunction,
                          int mappingUnit,
                          boolean rescale,
                          boolean skeleton)
```

:   Add a chromosome to the map cluster framework.

    :   **Parameters:**: `group` - the chromosome.: `crossDesign` - the cross design: `mappingFunction` - the mapping function.: `mappingUnit` - the mapping unit.: `rescale` - true if the chromosome must be rescaled: `skeleton` - true if the chromosome is the skeleton chromosome.

---


### getChromosomeName

```
public java.lang.String getChromosomeName(java.lang.String name)
```

:   This method returns the name of the chromosome
    from the given name. If no synonym dictionnary is defined
    then the method returns the same name.

    :   **Parameters:**: `name` - **Returns:**

---


### getClusters

```
public ChromCluster[] getClusters()
```

:   Returns the chromosome clusters. This method
    must be invoked after that all the maps have been
    added using the method `addMap(IBioGenome, boolean)`.
    Otherwise if any valid call has been done to this method
    then the returned array will be null.
     `IBioGenome[] mymaps = ...;
    MapCluster mapCluster = new MapCluster();
    for(i=0;i

    :   Returns:: an array of ChromCluster. See Also:: ChromCluster`

---


### setDubiousMarker

```
public void setDubiousMarker(IBioLocus[] dubious)
```

:   This method sets the list of dubious marker. Any call
    to the method `addMap(IBioGenome, boolean)` will
    then taken into account this list.

    :   **Parameters:**: `dubious` - the array of dubious loci. **See Also:**: `ChromCluster.setDubiousMarker(IBioLocus[])`

---


### getMarkerDico

```
public MetaDico getMarkerDico()
```

:   **Returns:**: Returns the mrkDico.

---


### setMarkerDico

```
public void setMarkerDico(MetaDico mrkDico)
```

:   **Parameters:**: `mrkDico` - The mrkDico to set.

---


### addAllMap

```
public void addAllMap(IBioGenome[] maps)
```

:   **Parameters:**: `maps` -


---


|  |  |  |
| --- | --- | --- |
| **Overview** \| **Package** \| **Class** \| **Use** \| **Tree** \| **Deprecated** \| **Index** \| **Help** | |  |
| **PREV CLASS**   **NEXT CLASS** | **FRAMES**    **NO FRAMES**     **All Classes** |  |
| SUMMARY: NESTED \| FIELD \| CONSTR \| METHOD | DETAIL: FIELD \| CONSTR \| METHOD |  |


---
